# Bacterial evolution in the oral microbiome: the role of conjugative elements and horizontal gene transfer

**DOI:** 10.1128/jb.00066-25

**Published:** 2025-07-01

**Authors:** Allison J. Renno, Robert C. Shields, Lisa K. McLellan

**Affiliations:** 1 Department of Biological Sciences, Purdue University Fort Wayne 14688, Fort Wayne, Indiana, USA; 2 Department of Biological Sciences, Arkansas State University 316447 , Jonesboro, Arkansas, USA; University of Massachusetts Chan Medical School, Worcester, Massachusetts, USA

**Keywords:** gene transfer, conjugation, oral microbiology, microbial communities

## Abstract

As one of the most diverse bacterial populations within the human body, the oral microbiome encodes a wealth of genetic information. Horizontal gene transfer, driven by mobile genetic elements, takes advantage of this information to influence bacterial evolution and the spread of phenotypes (antibiotic resistances, virulence attributes, and metabolic capabilities) among oral microbes. Although widespread within microbial communities, fundamental aspects of the mobile elements that drive horizontal gene transfer within the oral cavity remain poorly understood. In this review, we explore what is known about the role of horizontal gene transfer in bacterial evolution within the oral microbiome and the elements that facilitate this transfer, with a specific focus on conjugative DNA transfer. Conjugative elements are found in virtually all bacterial phylogenetic clades, and some can mediate genetic exchange between distantly related organisms. This is of particular interest in the diverse microcosm of the oral cavity, specifically how it drives the evolution and virulence of dental pathogens. Finally, we highlight advances in our understanding of the unique biology within dental plaque and how these might influence our understanding of bacterial gene transfer, and thus human health and disease.

## INTRODUCTION

The oral microbiome is an incredible example of a human-associated multi-species biofilm with a highly dynamic environment. Within the human oral cavity resides a complex and diverse ecosystem, composed of >700 unique bacterial species (and significantly more microbial species when considering fungi, viruses, and protozoa) (1). The oral microbiome plays a pivotal role in maintaining oral health while also being capable of contributing to disease (2, 3). The diversity of the oral microbiome can therefore be a key indicator of oral health. Reduced diversity can cascade into dysbiosis, often associated with disease states such as dental caries, periodontitis, and gingivitis (4–6).

Critical for bacterial survival in the oral cavity are mechanisms that provide both short-term adaptability and long-term evolution. Horizontal gene transfer (HGT) dominates microbial evolution—allowing bacteria to acquire genetic material from alternate sources rather than solely parental inheritance. The various pathways of HGT, including transformation, transduction, and conjugation (molecular mechanisms of HGT reviewed in Arnold et al. [7]), each play a unique role in shaping the genetic landscape of the oral microbiome, enhancing adaptability and survival of bacterial populations (Box 1). Although all types of HGT are incredibly important aspects of evolution in the oral microbiome, transformation is restricted to hosts with a certain degree of homology to the incoming DNA in order for the DNA to be integrated into the host genome (8). In contrast, conjugative elements can exhibit a broad host range and are often capable of mediating genetic exchange between more distantly related organisms (9, 10).

Box 1. **Types of horizontal gene transfer and notable examples within the oral cavity.**

- **Transformation:** Direct bacterial uptake of environmental DNA; incoming DNA must be sufficiently similar to the host genome to be incorporated into the recipient bacterial genome through homologous recombination
  - DNA source(s):
    - eDNA
    - Lysed cells
    - DNA may be from stable community members or from transiently passing bacteria
  - Notable examples: *Streptococcus, Haemophilus, Campylobacter, Neisseria,* and *P. gingivalis* (all naturally competent bacteria; transformation is a regular part of their lifestyle), as discussed in reference (11)
- **Transduction:** Viral-mediated DNA transfer; high viral load in the oral cavity, estimated at 10^8^ bacteriophages per 1 mL human saliva
  - DNA source(s):
    - Oral bacteriophages
  - Notable examples: Bacteriophages have been isolated or identified by metagenomic methods for many oral cavity species, including *Lactobacillus, Actinomyces, Aggregatibacter, Fusobacterium, Enterococcus, Porphyromonas,* and *Streptococcus* sp.—notable examples include (12, 13)
- **Fusion:** Process by which distinct membranes are brought into proximity of one another and merge to become one
  - DNA source(s):
    - Outer membrane vesicles
    - Another cell (protoplast fusion)
  - Notable examples: Many oral bacteria have been noted to produce vesicles (i.e., *Pseudomonas aeruginosa, Leptotrichia buccalis,* and *Streptococcus mutans*); however, in many cases, it is often unclear if these vesicles contribute directly to HGT and/or fusion, as discussed in references (14, 15)
- **Conjugation:** Direct method of DNA transfer from one bacterial cell (the donor) to another (the recipient) through cell-to-cell contact
  - DNA source(s):
    - neighboring bacteria (must be physically proximal for direct contact)
  - Notable oral cavity examples: Conjugative elements have been characterized or identified in all major clades of bacteria, and conjugative elements encoded within oral bacteria shown to experimentally transfer include Tn*916,* Tn*5397,* Tn*Smu1*, and CTnPg1 (16–20)

One of the most diverse bacterial populations within the human body is that of the oral cavity—second only to the diversity found within the colon (21). The complex bacterial communities present within dental plaque create unique opportunities for direct, interspecies bacterial interactions. This includes conjugative DNA transfer, potentially between genetically distinct oral bacteria. The role conjugation plays in bacterial evolution and diversity is therefore especially important to understand the context of the oral cavity—where close proximity and frequent interactions among bacterial cells further drive this mode of gene transfer (14).

With recent advances in genomics, large-scale sequencing of human microbiomes, and advanced microscopy techniques, we are now poised more than ever to begin to address the mechanisms of HGT that occur in the unique and complex environment of the oral cavity, specifically in how they relate to human health and disease. Here, we detail our present knowledge of HGT among oral microbes and highlight questions that are critical for our understanding of oral health and disease. Although all the mechanisms of HGT are briefly highlighted in relation to oral bacteria, the focus of this review is conjugation, as we believe the study of conjugative gene transfer may illuminate the unique biology and interactions that occur on a micron scale within the dynamic niche of the oral microbiome.

## A BRIEF INTRODUCTION TO HORIZONTAL GENE TRANSFER

Within prokaryotic cells of the oral cavity, HGT thrives in its three principal forms: transformation, transduction, and conjugation. The fourth type of HGT, a fusion between two cells or DNA-containing outer membrane vesicles, appears less common and thus is not a major focus of this review. Here and in Box 1, we briefly describe transformation, transduction, and conjugation. For further reading on the mechanisms of HGT, we suggest many of the excellent reviews on these topics, including, but not limited to, references (22–27).

Transformation allows for a type of scavenging in the microbial world—where bacteria sweep up free DNA from their environment (often the remnants of lysed neighbors) and stitch it into their own genomes through homologous recombination. Natural transformation is driven by competence machinery, encoded multi-protein complexes that allow for this DNA uptake. However, the impact transformation has is limited by homology requirements of the incoming DNA, strain-specific competence variability, and variable environmental signals that trigger competence development. For example, *Veillonella* species exhibit notable strain-to-strain variability in natural competence—some strains effectively take up DNA while in others competence is undetectable, as demonstrated by Knapp et al. (28).

Transduction, meanwhile, relies on bacteriophages as its couriers, packaging bacterial genes into viral capsids and delivering them to new hosts. There is great interest in the discovery and isolation of bacteriophages for basic biological purposes, their role in HGT, and also the treatment of various diseases through phage therapy. Although it is known via metagenomics that there are 10^8^ phages per 1 mL of saliva (29), phage often display a very narrow host range, often capable of infecting only specific species or even specific strains of bacterial hosts. Hence, although phages are highly prevalent, there have been limited studies of their role in ecology and their therapeutic potential in the oral cavity.

Conjugation is an intimate mechanism of HGT, requiring direct cell-to-cell contact in order to shuttle mobile genetic elements between donor and recipient cells. Conjugative DNA transfer occurs from one bacterial cell (the donor) to another (the recipient) through cell-to-cell contact. This process can involve plasmids (well-studied, described in reference [30]) and integrative and conjugative elements (ICEs), the most prevalent type of conjugative element (reviewed in reference [27]). Although the specifics of conjugation may vary based on the element and its specific bacterial host, for conjugation to occur, a donor cell containing the conjugative element must first encounter a permissive recipient cell. Encoded within the conjugative element is the necessary machinery, often a type four secretion system, to drive the conjugation process—including the formation of the transfer apparatus. In the event of a successful transfer, the recipient cell acquires this new genetic material, which can include genes for antibiotic resistance, metabolic capabilities, or virulence factors—all of which hold significance in the oral cavity (14).

## INFLUENCE OF ORAL BIOGEOGRAPHY ON CONJUGATION

Conjugation is influenced by environmental conditions and direct contact between the donor and recipient cells, altering the likelihood of horizontal gene transfer and the mobility of genetic elements. For these reasons, understanding both the physical environment and the interactions in the mouth—on both macro- and micro-scales—is essential for understanding conjugation. The oral cavity contains a variety of different surfaces that serve as complex habitats, each suiting the requirements and preferences of specific bacteria. The main components of the oral cavity are the tongue, teeth, gingiva, and the biofilms and microbial communities established on these surfaces. Although these environments are interconnected through salivary flow, each offers distinct physical and chemical niches, thus leading to adapted microbial communities that thrive in these specific surroundings. For example, fluorescence *in situ* hybridization (FISH) (and experimental derivatives of FISH thereof) has revealed complex and unique structures formed within the biofilms of the oral cavity (31–33). These highly structured communities are influenced by multiple factors including oxygen availability, pH, temperature, moisture, nutrient availability, adhesive surface structure and availability, and the shear force of salivary flow (31, 34–36). Therefore, the specific location within the oral cavity and the structural arrangement of biofilms influence the intergenus bacterial-bacterial interactions available and therefore the potential mating partners available to produce conjugative gene transfer (37).

Furthermore, numerous factors may influence the composition and diversity of microbial populations in the oral cavity. Diet is a major determinant of the composition of the oral microbiome (reviewed in reference [38]). Additionally, oral hygiene practices, including brushing, flossing, and the use of antimicrobial mouthwashes, can significantly reduce oral bacterial load and alter the composition of the oral microbiome (39–41). The microbes themselves are also major players in generating the structure of their own microbial communities. They can provide and metabolize nutrients and metabolites, generate and respond to antimicrobial molecules, and physically occupy space (either excluding or supporting other bacteria from that niche). Together, these factors form complex and diverse microbial biofilms, which create unique opportunities for gene transfer within these communities.

Building on these microbial interactions, the processes of HGT can be further influenced by the dynamics of bacterial growth. When bacteria proliferate, energy often goes to support growth and reproduction, which may alter their behavior in regard to HGT. For example, streptococci have been demonstrated to develop competence for genetic transformation during exponential growth due to the expression of a quorum sensing two-component system (42). In contrast, however, many oral bacterial genera are naturally competent at all times, regardless of growth stage (for example, *Neisseria and Campylobacter* sp.) (43). Similar trends are seen in conjugative elements. Some integrative and conjugative elements are activated, at least in part, by cell-to-cell signaling and growth phase (e.g., ICE*Bs1* from *Bacillus* sp [44]*,* Tn*916* from *Enterococcus*, *Clostridium*, *Streptococcus*, and *Staphylococcus* sp. [45]*,* and ICE*clc* from *Pseudomonas* sp. [46]), whereas others are activated independent of growth phases (e.g., Tn*Smu1* from *S. mutans* [18] and SXT from *V. cholerae* [47]).

Furthermore, many conjugative elements often respond to specific external stimuli in order to maximize the likelihood of HGT while minimizing the energy expenditure of the bacterial host; for example, the CTnDOT-ERL family of ICEs from *Bacteroides* sp. and Tn*916* display phenotypic-dependent induction in the presence of tetracycline (48–50). In some cases, even sugar substitutes may have some level of influence; *E. coli* donor and recipient strains exposed to different types of sweeteners have exhibited significant increases in conjugative transfer across various concentrations (51). Therefore, it is interesting to ponder whether specific location within the biofilm of dental plaque, or even external factors such as host diet and host medication, may influence conjugative transfer. Regardless of activation, however, if there is not a permissive recipient cell in physical proximity to the donor, conjugative transfer will not occur. Therefore, understanding the biogeography of bacterial communities on a micron scale is critical in understanding conjugative transfer that occurs in the oral cavity (described briefly below).

### The teeth

Teeth offer a hard, non-shedding surface ideal for adherence and biofilm formation. Initially, a thin film of glycoproteins called the salivary pellicle will coat the tooth surface, serving as a substrate for the initial attachment of oral microbiomes (52). Typically, the majority of bacteria that first colonize dental surfaces belong to the *Streptococcus* and *Actinomyces* groups, but as a biofilm matures, it expands and includes a variety of bacterial species (53). This biofilm of dental plaque grows to include *Corynebacterium, Streptococcus, Porphyromonas, Haemophilus, Aggregatibacter, Neisseriaceae, Actinomyces,* and *Rothia,* among others (31). Each of these microbes creates a unique niche that influences its nearby neighbors. For example, *Streptococcus* coating the tooth surface generates an oxygen-poor, carbon dioxide-rich environment where *Fusobacterium* filaments can proliferate and thrive (31). Furthermore, these biofilms are made up of a highly structured microbial community, which is embedded within a secreted matrix of extracellular polymeric substances (EPS) (54). Bacteria that possess virulence factors facilitating their survival within these biofilms typically have a competitive advantage. For example, *S. parasanguinis* FW213 contains five horizontally acquired genomic islands encoding bacteriocins, fimbriae, EPS biogenesis and export, and biofilm formation gene products, providing this organism an enhanced ability to compete and survive within oral biofilm (55). One major component in structural integrity is extracellular DNA (eDNA), which acts as scaffolding to hold the biofilm matrix together. High concentrations of eDNA in close proximity to bacterial cells increase the likelihood of genetic material being taken up via transformation (54, 56). However, for transformation to occur, the recipient cells require a significant degree of homology with the incoming DNA to be able to successfully incorporate it into its genome—limiting the range of bacteria capable of taking up this eDNA (43). In contrast, conjugative elements do not require homology for transfer; the host range of these elements is therefore often much larger (27). Furthermore, the close cell-to-cell contact within biofilms enhances the efficiency of conjugation, becoming a hotspot of horizontal gene transfer (57). Biofilms have been continuously shown to support high frequencies of horizontal gene transfer compared with free-living bacteria; it is an environment that promotes not only the physical interaction required for conjugation but also the stabilization and retention of transferred genes (58, 59). The formation and maintenance of these biofilms can be a central part of the process of bacterial conjugation, promoting interactions essential for the transfer of genes.

### The tongue

The tongue, covered in filiform papillae, serves as a highly structured and textured environment for microbial colonization. The microbial community here is relatively stable and is typically dominated by anaerobic bacteria such as *Veillonella* and *Streptococcus*—both of which can thrive in the low-oxygen environment that is created by the topography of the tongue’s surface (60). Furthermore, the mucosal surface of the tongue facilitates the growth of different species than the non-mucosal oral surfaces. For example, although *Rothia* and *Streptococcus* bacteria are both found on or within the tongue or dental plaque, *R. mucliaginosa* and *S. salivarius* appear to be more tongue-specific, whereas *R. aeria, R. dentocariosa*, and *S. sanguinis* are more plaque-specific (61). In contrast, some bacterial species are found in equal proportions throughout the tongue and plaque (i.e., *S. mitis* group) (61). Interestingly, the species on the tongue grow in a patch-like manner, with sections of the biofilm being occupied primarily by a single species, suggesting initial attachment and then a possible clonal expansion of the bacteria occupying that niche (61). Although HGT has not been extensively studied on the tongue surface, it is probable that distinct environmental conditions and the variation in the microbiome structure that is present can affect the frequency and nature of HGT on this site.

### The gingiva

The gingiva forms the interface between teeth and the soft tissues of the oral cavity. Plaque accumulation above the gumline and on tooth surfaces is defined as supragingival. On the other hand, subgingival plaque forms below the gumline and is a low-oxygen environment that supports primarily anaerobic bacteria (31, 62, 63). *Porphyromonas gingivalis* and *Treponema denticola* are two key species often found in this location, and when these bacteria proliferate in the gingival sulcus, it becomes a key site for the development of periodontal disease (63). These geographic regions contain unique environmental pressures compared with other areas of the oral cavity, and therefore bacteria residing here require different metabolic and virulence properties to thrive (64–68). For example, gingival crevicular fluid is an inflammatory exudate present in the subgingival crevice. Derived from periodontal tissues, this fluid—serum and protein-rich—is an important nutrient source for proteolytic bacteria, including *P. gingivalis* and *T. denticola* (69). Although HGT has not been extensively studied in the context of this specific surface, it is plausible that its unique environmental pressures may favor mobile elements that encode fitness advantages—perhaps elements encoding processes related to proteases, hemolysins, and iron-acquisition systems.

## MAJOR CONJUGATIVE ELEMENTS WITHIN THE ORAL MICROBIOME

Conjugative elements, particularly ICEs, are understudied and under-characterized, particularly in the context of the oral cavity. Genomic analysis suggests ICEs are highly prevalent within all major clades of bacteria, including those occupying the oral cavity (70). Recently, the ICE identification tool, ICEberg 3.0, extracted assembled sequences available from the Human Microbiome Project and bioinformatically identified 844 ICEs from 1,296 oral cavity samples (71). Furthermore, Lee et al. identified 173 ICEs and 233 integrated and mobilizable elements (IMEs, integrated elements often mobilized by other conjugative elements) throughout 551 publicly available Streptococcal genomes (both health- and disease-associated) (72). These elements were diverse in bioinformatically predicted cargo genes, suggesting that these elements play diverse functions in the oral microbiome. Similarly, 9 ICEs and 165 IMEs were identified within 75 genomes of *S. salivarius* (73)*,* and as more sequencing and functional characterization occurs, more ICEs and functional ICEs are identified (e.g., see Dahmane et al. [74]). As much of the work on oral conjugative elements has been through genomic analyses, here, we highlight experimentally characterized integrative and conjugative elements Tn*916* and Tn*Smu1*, along with several other notable (although less comprehensively studied) conjugative elements as examples of functionally characterized elements, what is known, and what remains to be discovered within oral conjugative elements.

### Tn*916*

Tn*916* is the most well-characterized ICE encoded in the bacteria of the oral cavity. Originally identified in *Enterococcus faecalis,* it possesses a broad host range—allowing it to transfer genetic information across a wide variety of bacterial species (75, 76). In general, the Tn*916* family of ICE elements represents a widespread and diverse family of related elements, often responsible for the dissemination of antibiotic resistances (most often to tetracyclines but including macrolides, lincosamides, and others) (16, 77). Tn*916* encodes for tetracycline resistance via the *tetM* gene (78). Notably, the *tetM* gene has been detected in up to seven different species of oral streptococci—including *S. mutans* and *S. oralis*—encoding resistance to tetracycline and its derivatives, such as minocycline (although it is unclear if in all these cases *tetM* was encoded on Tn*916* specifically, encoded elsewhere in the genome, or on other elements) (79). Furthermore, *Veillonella dispar* was shown to successfully transfer Tn*916* to four distinct *Streptococcus* species co-existing in a biofilm, facilitating the exchange of genes (11). Tn*916* DNA taken from *Veillonella dispar* was also capable of transforming streptococci species into tetracycline-resistant strains (11). This versatility in transfer methods of Tn*916* likely also contributes to the prevalence and persistence of this element through diverse bacterial species.

However, what we are now only beginning to appreciate is that many ICEs act as a double-edged sword to their host—contributing antibiotic resistance or other phenotypes to their host while also resulting in some sort of fitness defect (80). Tn*916* itself exhibits such a dual nature; while providing resistance to tetracycline, its excision can also trigger cell death in some cases. This highlights the need to study the complex interactions between ICEs and their hosts to generate a complete picture of the impacts of HGT within bacterial communities. Although the precise mechanism of Tn*916* host cell death still remains to be elucidated, the potential impacts on the fitness of the oral bacteria containing these conjugative elements and therefore their impact on the oral microbiome remain a fruitful area of study.

### Tn*Smu1*

Recently, we have characterized a novel ICE, Tn*Smu1* (18, 19). As opposed to Tn*916* with its broad host range, Tn*Smu1* appears to be primarily encoded within *Streptococcus mutans* strains (18, 19). Excision of Tn*Smu1* is prompted by DNA damage and growth on solid surfaces, potentially mimicking the environment *S. mutans* may encounter growing on a tooth surface (18, 19). However, unlike Tn*916*, excision of Tn*Smu1* does not result in host cell death but instead results in a cellular growth arrest, potentially limiting the immediate fitness costs associated with excision (18, 19). This arrest could represent an adaptive strategy to prevent the loss of Tn*Smu1* while this element is in its excised state. Although ICEs are capable of undergoing autonomous plasmid-like rolling circle replication (81, 82), when excised, Tn*Smu1* remains in low copy numbers (2–5 copies per cell) (18). By temporarily halting cell division during excision and transfer, Tn*Smu1* is more likely to ensure its inheritance within its host cell, thereby avoiding dilution in a growing population. The host cell may then be able to conserve energy that would otherwise be expended on the two ongoing processes—growth and conjugation—if growth were not otherwise halted. Alternatively, this growth arrest may simply reflect a fitness cost associated with Tn*Smu1* expression. This raises the possibility that Tn*Smu1* may encode some currently undiscovered fitness advantage that provides selective pressure to maintain the element despite this fitness disadvantage. However, any advantageous phenotypes encoded by Tn*Smu1* remain to be identified, reflecting the continued need for study and understanding of these elements.

For ICEs like Tn*916* and Tn*Smu1,* it is interesting to speculate how the widespread use of antimicrobial agents in oral healthcare products may influence ICE spread. Common treatments like hydrogen peroxide-based whitening products or antiseptic mouthwashes may induce DNA damage without necessarily achieving bactericidal concentrations (83, 84). As DNA damage is a common trigger for the excision of mobile genetic elements, this may paradoxically trigger excision and potentially accelerate the spread of mobile genetic elements in the oral cavity, and any phenotypes encoded within.

### Other notable elements

In addition to Tn*916* and Tn*Smu1*, several other functional conjugative elements have been identified within the oral microbiome. One such element is CTnPg1, an ICE found within *P. gingivalis*, which has been implicated in the horizontal transfer of antibiotic-resistance genes, particularly those conferring resistance to tetracycline and erythromycin (85, 86). CTnPg1 is not limited to *P. gingivalis* but has also been detected in other oral bacteria, including *Bacteroides thetaiotaomicron*, *Porphyromonas endodontalis,* and *Prevotella* sp. (85, 86). Its wide distribution among oral anaerobic gram-negative bacteria could suggest an important role in promoting genetic exchange with oral microbes in close proximity in the oral cavity.

Other notable oral conjugative elements include CTn6002, Tn*5397*, and pAM81, each with distinct characteristics and implications in the spread of antimicrobial resistance. CTn6002, an ICE suggested to be involved in the spread of resistance to doxycycline and erythromycin, is encoded within *S. oralis* (87). Tn*5397*, originally identified in *Clostridium difficile*, has been observed to successfully transfer between species and to oral streptococci in biofilms (17, 88). Similar to Tn*916* in its ability to spread tetracycline resistance, Tn*5397* may have a role in the dissemination of resistance traits between species within the oral cavity (88, 89). Its functional similarity to Tn*916*, particularly in spreading tetracycline resistance, underscores its potential role in the interspecies exchange of resistance traits in the oral microbiome.

Adding to this diversity in mobile genetic elements is pAM81, a conjugative plasmid found in oral *Streptococcus* and *Enterococcus* species and known to facilitate the transfer of erythromycin resistance (90). Although plasmids like pAM81 are not integrated into the host genome, their ability to mobilize resistance genes remains important within the oral microbiome, potentially driving traits for survival (90). Together, this highlights the diversity of elements within the oral microbiome and their potential role in the virulence attributes of their host. However, this is only a small glimpse into the variety of conjugative elements in the oral cavity, as countless others remain to be discovered and characterized.

## HORIZONTAL GENE TRANSFER AND DEFENSE MECHANISMS SHAPE AND INFLUENCE EACH OTHER

HGT is not a one-sided affair. Bacterial defense mechanisms (e.g., restriction-modification and CRISPR-Cas systems) can protect host cells from and lessen risks posed by incoming mobile genetic elements. Although these systems were historically viewed as defenses primarily against bacteriophages, they also target a wide range of mobile genetic elements. For example, although CRISPR arrays in oral bacteria indeed contain spacers targeting bacteriophages, CRISPR spacers have been found to target conserved regions of ICEs, including the DNA processing and conjugation machinery necessary for their function (91, 92). For instance, CRISPR spacers against Tn*Smu1* are common among *S. mutans* strains (93). Across different human microbiomes, the oral microbiome was found to exhibit one of the highest CRISPR spacer loads, reflecting the high diversity of genetic material present and passing through this niche (94). These systems can function as a “molecular tape recorder,” capturing a history of past invasions by integrating spacers from any foreign DNA into the CRISPR array and the evolutionary arms race that occurred from there.

Notably, mobile genetic elements themselves carry a variety of defense systems against other mobile genetic elements, adding further complexity to bacterial interactions with mobile DNA. For example, in Tn*916* host *B. subtilis,* Tn*916* activation leads to lethality. This lethality is caused by interactions with the Tn*916* conjugation machinery and a gene encoded by a separate mobile genetic element, phage-like *sigK* intervening element (*skin*)-encoded *yqaR* (although the mechanism in other Tn*916* hosts is more complex) (45). Additionally, other conjugation systems may rely on pili, which serve as targets of male-specific bacteriophages, in some cases completely blocking conjugation (95–100). Although not an oral bacterium, another notable example of ICE/phage interactions is the *B. subtilis* ICE, ICE*Bs1*. ICE*Bs1* defends against a specific phage via an abortive infection system, effectively halting the phage’s spread (101). Interestingly, in a screen in *E. coli* for phage defense systems, most anti-phage defense systems were found on other mobile genetic elements, again highlighting the complex relationships that mobile genetic elements share with their host and each other (102, 103). However, although these systems defend against unwanted genetic material, they may also limit beneficial gene acquisition.

However, not all interactions between mobile genetic elements are antagonistic. Many transposons and plasmids that do not encode machinery for self-transfer can be transferred to new hosts by conjugation machinery encoded by a co-resident ICE. Additionally, as ICEs are modular in nature, integration next to other mobile genetic elements may create composite elements and tandem arrays, generating diversity in the ICEs and the genomic material that may then be transferred (27, 104). Additionally, RNA-seq analysis has shown that in *S. mutans,* the activation of Tn*Smu1* leads to the upregulation of not only its own genes but also that of the type-I-C CRISPR-Cas system (19). This simultaneous upregulation may suggest a coordinated response, where activation of the ICE may trigger CRISPR-Cas activity to potentially serve as a defense mechanism against the risks associated with mobilization of Tn*Smu1*, regulate the activity of the ICE itself, or function as a protective measure against other incoming mobile genetic elements. Together, this begs the age-old question: How do bacteria balance between defense and adaptability in bacterial communities?

## ROLE OF CONJUGATION AND HGT IN ORAL HEALTH AND DISEASE

Oral diseases (e.g., dental caries, periodontitis) and systemic diseases (e.g., infective endocarditis) caused by oral bacterial pathogens—including *S. mutans*, *P. gingivalis,* and *A. actinomycetemcomitans*—are a major global health concern. The ability of these pathogens to evolve rapidly through horizontal gene transfer only further exacerbates the challenge. As the impact of HGT on oral health and disease is understudied, it is hard to quantify the specific impact of these processes. However, what we do know is that the gain of genes via HGT and reductions through gene loss cause significant genomic heterogeneity between pathogenic strains. For example—and remarkably—S. *mutans* strains diverge by 100–300 genes on average, resulting in an estimated pangenome of ~15,000 genes (93, 105). This genomic variability can complicate treatment strategies, as the *S. mutans* population in one individual’s mouth may harbor vastly different virulence factors and resistance genes compared with another’s—making standardized therapies less effective.

Additionally, conjugative elements promoting the evolution of pathogenic strains through their encoded cargo could have vast effects on the health of the oral cavity, increasing the severity and persistence of oral diseases. For example, some oral *Streptococcus* sp. have been shown to harbor several resistance genes linked to multiple antibiotics—compounding the spread of antibiotic resistance, particularly within the oral biofilms dominated by this species (106). Analysis of bioinformatically identified conjugative elements in oral streptococci identified nearly 2,000 virulence genes from a total of 200 conjugative elements. These included effector delivery systems, immune modulators, nutritional/metabolic factors, adherence factors, and exotoxins (72). Genes that alter immune responses can interfere with the inflammatory and immune signaling pathways in the oral cavity—again impacting the host’s ability to maintain a balanced, healthy ecosystem and potentially leading to a recurring dysbiosis of the oral microbiome (107–109).

Furthermore, the overuse of antibiotics—whether in clinical or agricultural settings—can have ecological consequences outside of the intended target. Tetracycline, for example, is shed via the urine and feces of animals and humans, resulting in environmental contamination and thus the development of tetracycline-resistant microbes (110). Similarly, in the oral cavity, it is interesting to ponder whether the excessive or even improper use of antibiotics or mouthwash antiseptics may inadvertently continue to promote HGT, not only among oral bacteria but also others within the microbiome, as these microbes may transiently colonize other body sites (e.g., travel through the throat and interactions with disease- and health-associated bacteria present). As a consequence, there remains the increasing possibility of the emergence and dissemination of highly virulent and multi-drug resistant strains through HGT, both within the human mouth and in other ecosystems.

Furthermore, HGT can impact both the long-term evolution and short-term heterogeneity of microbes. There is evidence that *S. mutans* virulence traits, sugar metabolisms, and pH tolerance genes (that are absent in other Mutans group streptococci) were horizontally acquired from other lactic acid bacteria when humans first began advanced agriculture (111). Additionally, HGT mechanisms generate short-term heterogeneity that is intimately connected with physiology. This is evident for conjugative elements in the change in physiology seen when Tn*916* and Tn*Smu1* are activated (see **Major conjugative elements within the oral microbiome**). There is also a strong connection between *S. mutans* virulence and the transient physiological state generated during genetic competence. Notably, strains that are defective in genetic competence are also defective in stress tolerance and biofilm formation (42, 112). We are only beginning to appreciate the deep connections between HGT and virulence in oral bacteria. Thus, it is paramount to understand the various mechanisms of HGT and its impact on host cell physiology and virulence.

## CONCLUDING REMARKS

Over the last two decades, genome sequencing has highlighted the profound diversity within the bacterial species of the oral microbiome. Much of the genomic diversity that the scientific community has observed is likely generated via HGT events, of which ICEs and related elements form a considerable portion. However, our knowledge of these elements, and the genes they carry, is in its infancy, and there are countless elements for which we have no evolutionary or functional knowledge. It is a complicated truth that most of our knowledge of oral bacteria has been focused on laboratory strains, which do not fully reflect the natural diversity and repertoire of phenotypic traits carried by wild isolates. To understand the genetic repertoire of wild isolates, their fluidity, and ultimately how their phenotypic traits influence oral health and disease, we have to develop a greater understanding of horizontal gene transfer and conjugative elements within the oral cavity.

**Fig 1:**
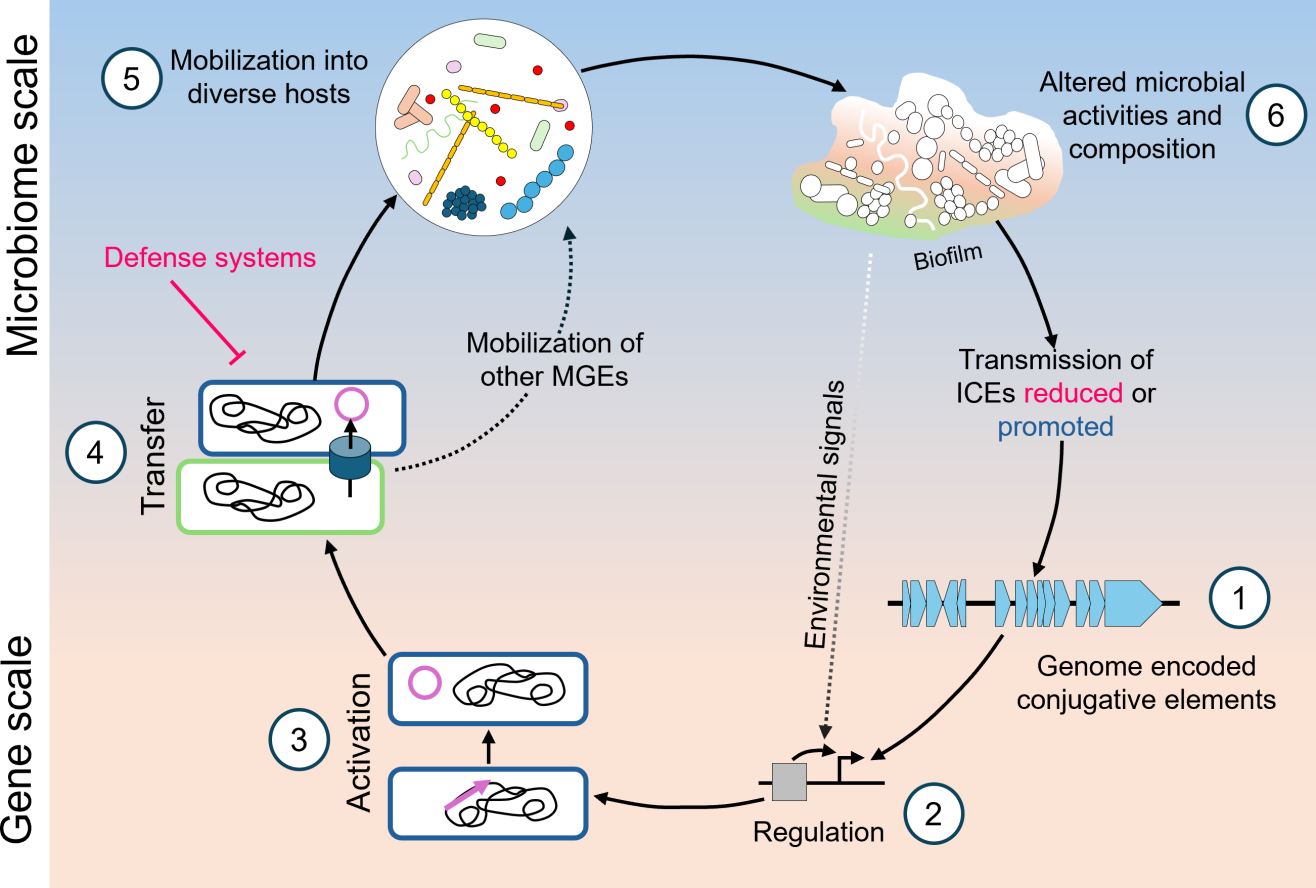
Proposed model for the impact of conjugative elements on oral microbiome activity and composition. Within a host organism, genome-encoded conjugative elements (1), including integrative and conjugative elements (ICEs) and integrative and mobilizable elements (IMEs), respond to environmental signals that regulate their activation (2). Once active (3), these elements can transfer between cells if an activated donor cell encounters a permissive recipient (4). Furthermore, these elements may mobilize other mobile genetic elements (plasmids, genomic islands, and other ICEs and IMEs), furthering the genetic transfer that is occurring on the microbiome scale. However, during this process, conjugative elements may encounter bacterial defense mechanisms (e.g., CRISPR, restriction-modification systems, and exclusion systems), which can inhibit or prevent transfer. As many conjugative elements have a broad host range in which they can transfer, these elements can spread to diverse microbial populations (5), altering microbial activity and reshaping microbiome composition (6). These changes may, in turn, modify environmental signals, the pool of available recipient cells, and other factors, perpetuating a dynamic cycle in which conjugative elements continuously influence the function and composition of the oral microbiome.

To generate a comprehensive understanding of conjugative elements, there are two scales that the scientific community must investigate—micro and macro (Fig. 1). Our understanding at the micro-level begins with genomic sequencing and the identification of ICEs and related elements. Advances in generative artificial intelligence and high-performance computing clusters will likely improve computational identification of these elements, particularly in large metagenomics data sets. That being said, the computational discovery of elements has little bearing on our functional knowledge of them. This is where basic research into the mechanisms of conjugative elements is required, including regulation, transfer, host range, and impacts on host cell physiology. It is currently unknown how many of the computationally discovered elements are capable of conjugative transfer and into what range of bacterial species. Broad and unbiased tracking of newly discovered elements may be feasible with microscopic (e.g., FISH or fluorescent sensors) and genetic/genomic (e.g., CRISPR molecular recorders) approaches. At the macro, or microbiome, level, the ultimate goal is to understand how ICEs and conjugative elements influence the evolutionary trajectory of oral bacteria and how this, in turn, might then influence community scale dynamics. It is well known that oral diseases, such as dental caries, are driven by changes in bacterial species richness and abundance that stem from environmental pressures. By integrating and stably altering genomes, ICEs are perfectly positioned to help (or potentially hinder) bacterial survival as the community and environment are modified. Mechanistic studies at this scale have inherent challenges; as such, foundational and model strain data are necessary to build the tools to understand systematically if and how conjugative elements alter disease epidemiology and health outcomes. Understanding the role of conjugative elements in bacterial adaptation could ultimately inform novel approaches to mitigating the genetic drivers of oral diseases. With a deeper understanding of conjugative elements and horizontal gene transfer, we are poised to reveal deep truths of microbial evolution, community dynamics, and ultimately dental health within the oral cavity.
